# Leveraging the power of partnerships: spreading the vision for a population health care delivery model in western Kenya

**DOI:** 10.1186/s12992-018-0366-5

**Published:** 2018-05-08

**Authors:** Tim Mercer, Adrian Gardner, Benjamin Andama, Cleophas Chesoli, Astrid Christoffersen-Deb, Jonathan Dick, Robert Einterz, Nick Gray, Sylvester Kimaiyo, Jemima Kamano, Beryl Maritim, Kirk Morehead, Sonak Pastakia, Laura Ruhl, Julia Songok, Jeremiah Laktabai

**Affiliations:** 1 Department of Population Health, The University of Texas at Austin Dell Medical School, 1701 Trinity St, Austin, TX 78712 USA; 2 Department of Medicine, Indiana University School of Medicine, 535 Barnhill Dr, Indianapolis, IN 46202 USA; 3 Department of Medicine, Moi University School of Medicine, PO Box 4606 30100, Eldoret, Kenya; 4 Academic Model Providing Access to Health Care (AMPATH), PO Box 4606 30100, Eldoret, Kenya; 5 Department of Obstetrics and Gynaecology, University of Toronto Faculty of Medicine, 123 Edward Street, Suite 1200, Toronto, ON M5G1E2 Canada; 6 Department of Reproductive Health, Moi University School of Medicine, Eldoret, Kenya; 7 Dow AgroSciences, 9330 Zionsville Rd, Indianapolis, IN 46268 USA; 8 Purdue University College of Pharmacy, 575 Stadium Mall Dr, West Lafayette, IN 47907 USA; 9 Department of Pharmacology, Moi University School of Medicine, Eldoret, Kenya; 10 Department of Pediatrics, Indiana University School of Medicine, 705 Riley Hospital Dr, Indianapolis, IN 46202 USA; 11 Department of Child Health and Paediatrics, Moi University School of Medicine, PO Box 4606 30100, Eldoret, Kenya; 12 Department of Family Medicine, Moi University School of Medicine, PO Box 4606 30100, Eldoret, Kenya

**Keywords:** Population health, Global Health, Health care delivery system, Vision, Strategy, Partnerships, Kenya, Low- and middle-income countries (LMICs)

## Abstract

**Background:**

The Academic Model Providing Access to Healthcare (AMPATH) has been a model academic partnership in global health for nearly three decades, leveraging the power of a public-sector academic medical center and the tripartite academic mission – service, education, and research – to the challenges of delivering health care in a low-income setting. Drawing our mandate from the health needs of the population, we have scaled up service delivery for HIV care, and over the last decade, expanded our focus on non-communicable chronic diseases, health system strengthening, and population health more broadly. Success of such a transformative endeavor requires new partnerships, as well as a unification of vision and alignment of strategy among all partners involved.

*Leveraging the Power of Partnerships and Spreading the Vision for Population Health.*

We describe how AMPATH built on its collective experience as an academic partnership to support the public-sector health care system, with a major focus on scaling up HIV care in western Kenya, to a system poised to take responsibility for the health of an entire population. We highlight global trends and local contextual factors that led to the genesis of this new vision, and then describe the key tenets of AMPATH’s population health care delivery model: comprehensive, integrated, community-centered, and financially sustainable with a path to universal health coverage. Finally, we share how AMPATH partnered with strategic planning and change management experts from the private sector to use a novel approach called a ‘Learning Map®’ to collaboratively develop and share a vision of population health, and achieve strategic alignment with key stakeholders at all levels of the public-sector health system in western Kenya.

**Conclusion:**

We describe how AMPATH has leveraged the power of partnerships to move beyond the traditional disease-specific silos in global health to a model focused on health systems strengthening and population health. Furthermore, we highlight a novel, collaborative tool to communicate our vision and achieve strategic alignment among stakeholders at all levels of the health system. We hope this paper can serve as a roadmap for other global health partners to develop and share transformative visions for improving population health globally.

## Background

Global health partnerships are critical for transforming the landscape of service delivery in low-income settings [1]. The Academic Model Providing Access to Healthcare (AMPATH) is an academic partnership between Moi University and Moi Teaching and Referral Hospital (MTRH), a medical school and public-sector national teaching and referral hospital in western Kenya, and a consortium of North American academic medical centers led by Indiana University [2, 3]. The partnership began in 1989, as Moi University School of Medicine enrolled its first class of students, with a commitment to unleashing the inherent power of a Kenyan academic medical center to develop leaders in health care and to support and improve the Ministry of Health (MOH) care system. AMPATH strives to serve as an innovative engine of the Ministry of Health (MOH) in western Kenya, drawing its mandate from the health needs of the population. This academic model, unlike many other public-private partnerships or non-governmental organizations, brings to bear the power of the tripartite academic mission – service, education, and research – to the challenges of delivering population-based health care in a low-income setting. From the inception of the partnership, the focus was on supporting Moi University School of Medicine, MTRH, and the Kenyan MOH in embodying the post-Alma Ata principles of primary health care. Starting in 2001, AMPATH’s efforts shifted to focus largely on the prevention, treatment and control of the HIV epidemic in Kenya, as one of the largest implementing partners of the President’s Emergency Plan for AIDS Relief (PEPFAR) in sub-Saharan Africa [4–6]. As the HIV care program grew, AMPATH leveraged these investments in leadership, infrastructure, supply chains, information systems, training, and workforce to address non-communicable chronic diseases, strengthen the public-sector health system, tackle poverty and other health inequities, and develop community-centered approaches to population health more broadly. Please see Table 1 which highlights AMPATH’s accomplishments in service delivery over the years.

**Table 1:** Responding to Population Health Needs: AMPATH Accomplishments in Service Delivery

**Institutional Partnership Building 1989–2001**	• Moi University School of Medicine curriculum is developed, with a focus on rural and community-based health care, embodied in their Community-Based Education and Service (COBES) program • Indiana University (IU) initially commits to supporting Moi University in primary health care, COBES, and curriculum development. IU embodies this commitment with a full-time faculty member on ground in Kenya at Moi University every year • With support from Indiana University, new surgical suites and surgical care capacity is built at Moi Teaching and Referral Hospital • The platform for bilateral exchange is created; IU and other North American students, residents and faculty rotate in Kenya, and Moi students, residents (registrars), and faculty rotate and train at AMPATH institutions in North America • In response to population health needs and health system challenges, new North American academic partners join the consortium; by 2018, there are a total of 14 North American academic partners involved
**HIV Era 2001 – present**	• In 2001, the first patient at MTRH is treated with anti-retroviral therapy, launching AMPATH (The Academic Model for the Prevention and Treatment of HIV/AIDS). Over the years, AMPATH has received three cycles of USAID-PEPFAR funding for HIV care, with over 150,000 patients enrolled and 85,000 active on anti-retroviral therapy delivered at nearly 500 MOH-supported clinics across western Kenya. Moi University is the first African institution to be the prime grantee recipient of this type of funding • With support from Brown University, community and hospital-based programs for tuberculosis (TB) care, training, and research is developed • AMPATH partners with the World Food Program to support nutrition for patients with HIV • Kenya’s first EMR, the AMPATH Medical Record System (AMRS) is developed at a single HIV clinic, scaled to all clinics across the catchment area, and developed into OpenMRS which is now in use in over 60 countries worldwide • AMPATH launches home-based counseling and testing “HCT” to go door-to-door testing for HIV at every home in the catchment area, eventually reaching nearly 2 million households • Programs to support orphans and vulnerable children (OVCs) and address food and income security are developed alongside the HIV care program • The Legal Aid Centre of Eldoret (LACE), a human rights law center for HIV-infected patients is developed
**Population Health Era 2010 – Present**	• AMPATH changes its name to reflect its broader population health mission, now called The Academic Model Providing Access to Healthcare • Hypertension and diabetes screening are layered onto the HIV home-based counseling and testing program • The chronic disease management program is launched, focusing on hypertension and diabetes, providing care for nearly 15,000 patients at 69 MOH-supported facilities • With support from Indiana University, pharmaceutical industry partners, and the NIH, the Center of Excellence in Oncology was created, providing clinical care, training, and research for cancer • With support from Duke University, philanthropic partners, and the NIH, the Center of Excellence for Cardiovascular and Pulmonary Disease Research was created, along with the cardiac care unit (CCU) at MTRH, Kenya’s first in the public sector, providing clinical care, training, and research for cardiovascular diseases • With support from the University of Toronto, Indiana University, philanthropic partners, and Grand Challenges Canada, a community-based, maternal-child health program is developed. In addition, Riley Mother Baby Hospital at MTRH is built, dedicated to clinical care, training, and research for pregnant mothers and newborns • Purdue University commits full-time faculty members on the ground in Kenya, who, working with Moi University faculty, develop innovative and financially sustainable supply chain models for chronic disease medicines, and have pioneered the training of a new pharmacy workforce in Kenya • With support from the Regenstrief Institute at Indiana University, and faculty at Brown University, informatics and data science capacity is created, creating robust data infrastructure, biostatistics training, and expanded functionality of the AMRS EMR • With partnership from Dow AgroSciences and Purdue University, agricultural training and farm development support nutrition and income generation for Kenyan families, and in turn become a food distributor to the World Food Program • GISE (Group Integrated Savings for Empowerment), a community-based savings and loan program is launched. Following this, BIGPIC (Bridging Income Generation with Group Integrated Care) is created, coupling chronic disease management for hypertension and diabetes, to these microfinance GISE groups. AMPATH investigators subsequently received NIH-funding to evaluate this model of care delivery • The Chandaria Cancer and Chronic Disease Center is opened at MTRH, Kenya’s first dedicated center in the public sector to support clinical care, teaching and research for cancer and chronic disease • AMPATH, working within the Kenyan MOH, is working to secure a partnership with Kenya’s National Hospital Insurance Fund (NHIF) to be a principal service provider for primary care in the public sector in western Kenya. • AMPATH’s vision for Population Health is spread with the Learning Map® tool, and work ramps up towards a comprehensive, integrated, community-centered, and financially sustainable health care delivery system responsive to the needs of an entire population

Leveraging the power of partnerships that is inherent in AMPATH’s model, as well as building new ones, is critical to succeeding in this endeavor. Equally important is unifying all partners around a common vision, rapidly spreading that vision to key stakeholders, and achieving strategic alignment among all partners involved [7–9]. Additional academic partners from North America have joined the consortium over the years to address these additional health system challenges, driven by a common goal to work alongside colleagues at Moi University and MTRH, and within the Kenyan MOH care system, to help build capacity in service delivery, education, and research. AMPATH’s success is explicitly drawn from our institutional partnership model. A fundamental principle of this partnership is that academic institution and health system strengthening are built on the integrity of mutually beneficial and mutually respectful individual counterpart relationships between North Americans and Kenyans at all levels. This partnership is embodied in an African Proverb that is at the core of our work: “If you want to walk fast, walk alone. If you want to walk far, walk together.” The work of the AMPATH partnership is administered by an executive leadership team consisting of the Principal of Moi University College of Health Sciences (MUCHS), the CEO of Moi Teaching and Referral Hospital (MTRH), and the Executive Field Director of AMPATH at Indiana University, representing the North American partners. It is primarily executed by the long-term commitment of North American faculty presence on the ground in Kenya, working in partnership with colleagues and staff at Moi University and MTRH, and further facilitated by monthly consortium-wide conference calls, annual consortium-wide in-person meetings, and countless informal means of electronic and in-person communication.

## Laying the groundwork for a population health care delivery model in western Kenya: Global trends and local contextual factors

**Fig. 1:**
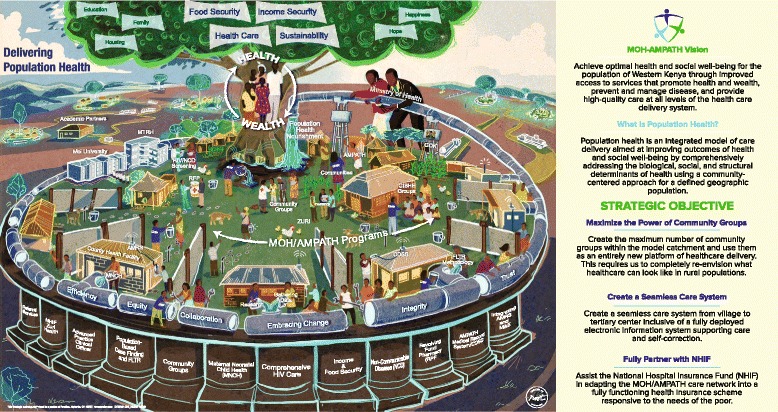
MOH-AMPATH Population Health Care Delivery Model

Nearly every public-sector health care delivery system in sub-Saharan Africa (SSA), including Kenya, faces a familiar challenge – an enormous disease burden, widespread poverty, an insufficient health care provider workforce, and a woefully inadequate proportion of the national budget designated for health services [10–15]. Despite these enormous challenges, public-sector health care delivery systems, international donors, and implementing partners alike have not been idle. Significant progress has been made across SSA in combating HIV, reducing maternal and infant mortality, and addressing non-communicable diseases (NCDs) like hypertension and diabetes [16–19]. But too often these responses are implemented in disease-specific silos – “vertical” programs that leave other key components of the care system behind [20, 21]. AMPATH, in cooperation with the Kenyan MOH, is taking a different approach. Recognizing the profound potential of the health system infrastructure created by global funding for HIV/AIDs, AMPATH and the Kenyan MOH are building a revolutionary new model of population health – a comprehensive, integrated, community-centered, and financially sustainable health care delivery system responsive to the needs of an entire population **(**Fig. 1**)**.

Population health is defined differently by different stakeholders in different contexts [22–26]. We define population health as a responsive, equitable, and integrated system of service delivery, inclusive of health promotion, disease prevention, treatment, rehabilitation, and palliation, aimed at improving health by comprehensively addressing the biological, social, and structural determinants of health using a community-centered approach for a defined geographic population. This definition comes in response to several historical trends in global health. First, is the “vertical”, siloed approach of disease-specific, donor-funded programs that abnegate responsibility for building a strong, comprehensive, public-sector health system [20, 21, 27]. Second, is the limited focus on social and structural determinants of health, and poverty reduction strategies, in the design of health interventions [28, 29]. And third, is the passive, patient-initiated, facility-based model of care that is pervasive throughout the world [30, 31].

From 2001 to 2010, AMPATH stood for the Academic Model for the Prevention and Treatment of HIV/AIDS [4]. In 2010, AMPATH changed its name to stand for the Academic Model Providing Access to Healthcare, which, in reality, brought us back to our original vision of unleashing the power of an academic medical center to respond to the comprehensive health needs of the population it serves. After several years of setting up the health system infrastructure to deliver HIV care, five central themes emerged that spoke loudly to the need for a comprehensive, integrated, community-centered, and financially sustainable population health care delivery model. First, there was a significant chronic disease burden other than just HIV [10, 32]. Second, the existing health system infrastructure was fragmented, weak, and ill-equipped to manage chronic illness across the disease continuum and throughout the life course [27, 30]. Third, there was insufficient activity designed to promote health and prevent disease at the community level [33–36]. Fourth, health sector activities were poorly responsive to the broader socioeconomic determinants of health, and limited in scope in their efforts to address poverty as a fundamental cause of health inequities [28, 37–39]. And fifth, a pathway to universal health coverage was needed as perpetual donor-funded models of care were unsustainable [40–43]. The time was ripe to move forward with a model of population health care delivery, and we were presented with three important, and timely opportunities. First, Kenya had recently undergone “devolution”, decentralizing the health sector to each of Kenya’s 47 counties, resulting in a potentially more responsive and lean MOH partner, committed to the needs of local populations [44–46]. Second, Kenya’s National Hospital Insurance Fund (NHIF) had recently launched its “SupaCover” program, the first government-backed outpatient, primary care insurance program in the country, creating a pathway towards universal health coverage for the population [47]. Third, we had the opportunity to leverage the leadership, infrastructure, supply chains, information system, workforce, and service delivery mechanisms that had been developed through the PEPFAR investment in Kenya, transforming an HIV care platform into one poised to tackle chronic disease and population health [48–50].

### AMPATH’s population health care delivery model

Over the years, AMPATH has been building many of the necessary components of a comprehensive population health model (see Table 1**)**. These include clinical programs covering adult and pediatric HIV, prevention of mother-to-child transmission of HIV (pMTCT), maternal, neonatal, and child health (MNCH), family planning (FP), tuberculosis (TB), diabetes, hypertension, cardiovascular disease, anticoagulation, oncology, palliative care, and mental health, as well as several cross-cutting departments including human resources and administration, grants management, pharmacy, laboratory, informatics, food and income security, monitoring and evaluation, quality improvement, and research [4–6, 18, 19, 38, 39, 51–74]. Kenya’s first electronic medical record system, the AMPATH medical record system (AMRS), was initially built to support care at a single rural health center, but has now grown to support HIV, MNCH, and NCD care, as well as monitoring and evaluation, quality improvement, and research, across much of AMPATH’s catchment area. Building on the HIV clinical infrastructure, AMPATH’s chronic disease management program now cares for thousands of patients with hypertension and diabetes at MOH-supported facilities across multiple counties, coupled with advanced cardiovascular care services at MTRH. Similarly, in both MNCH and oncology, community-based screening, care and workforce training have been coupled with advanced diagnostic and treatment services at MTRH. This has become a common pattern of coupling community-based care with specialty, hospital-based services, spanning across the tripartite academic mission of service, teaching and research. As the program has grown, however, the activities of these different components have, at times, become disjointed. The potential to unify our strategy and realize the synergies that existed was upon us. To build a population health care delivery model, AMPATH is breaking down these silos and integrating all of the component elements across each of the World Health Organization’s (WHO) health system building blocks – leadership and governance, financing, workforce, medicines and technologies, information systems, and service delivery [75]. Not only will the care system require integration across the health system building blocks, but service delivery points will require integration from primary to tertiary levels of care, connected by a seamless electronic health information system, such that the health system is designed to manage disease, and promote health, throughout the life course [27]. A population health care delivery model must also be more responsive to, engaged with, and driven by members of the community. A community-centered model of care involves getting out of the clinic and meeting the people where they are, delivering health prevention, promotion and care services in the home or other community spaces, such as workplaces, schools, and among community groups or gatherings [30]. Inherent in this approach is a proactive responsibility to promote health, and not just react to disease. This involves listening to the peoples’ voices in defining their own health priorities and holding their care system accountable to ensure access, quality, equity and the fulfilment of their right to health and social well-being. Lastly, a population health care delivery model requires mechanisms to protect the population from the impoverishing financial consequences of illness, and deliberately work to ensure food and income security. By leveraging microfinance groups as platforms for health promotion and care delivery, and through new partnerships with Kenya’s NHIF, AMPATH has the potential to develop a sustainable, domestic financing mechanism that will ensure universal health coverage for the entire population of western Kenya [76]. Currently AMPATH supplements Kenyan MOH financing of its health care system through a combination of individual philanthropy, private and corporate foundations, and U.S. government funding through PEPFAR-USAID. Our hope is that, over time, the need for external donor funding will decrease as sustainable, domestic financing mechanisms are scaled up.

### Spreading the vision through the power of partnerships

Dow AgroSciences is a corporate partner with AMPATH, helping build capacity for our agricultural and nutrition programs that support our comprehensive care delivery model. Starting in November 2015, AMPATH partnered with two strategic operations and change management experts from Dow AgroSciences, who had previously worked with the change management consulting firm called Root Inc. Root Inc. has helped many Fortune 500 companies, including Dow, to create breakthrough change in their organizational mission, culture, and strategy. Leveraging this pre-existing partnership with Dow AgroSciences, we were able to engage the change management expertise and tools developed by Root Inc. to help develop our vision and strategy for building a population health care delivery model in western Kenya. Root Inc. uses a unique methodology called a ‘Learning Map®’ visual that represents the vision and strategy through a detailed infographic and conceptual illustration **(**Fig. 1**)**. This Root Learning Map® module then becomes a metaphor for telling the story of the vision throughout an organization, rapidly creating a common understanding, shared language, alignment of priorities, and an intellectual and emotional connection to the strategy. The Learning Map®, along with supporting data, engaging questions and a facilitated discussion guide, unleashes the power of large groups of people to gain clarity of vision to catalyze change [77, 78].

The development of the Learning Map® started with several conference-calls over a four-month period between an internal planning and leadership team at AMPATH in Kenya, the strategic operations and change management experts from Dow AgroSciences, and a team of consultants and artists from Root Inc. During these meetings, we explained and refined AMPATH’s vision and strategy for population health through a series of sequential, facilitated discussions. These discussions covered the mission and goals of AMPATH; the history and accomplishments of the existing AMPATH-MOH care delivery system; how the components and programs of AMPATH come together to deliver on the vision; the strategic objectives and priorities to achieve the vision; the building blocks within the existing programs that need to be leveraged to realize the vision; and the important internal and external stakeholders, their roles, and the behavior changes needed to make the vision a reality. Through this series of discussions, the Learning Map® was iteratively created, and the discussion guide was built.

**Fig. 2:**
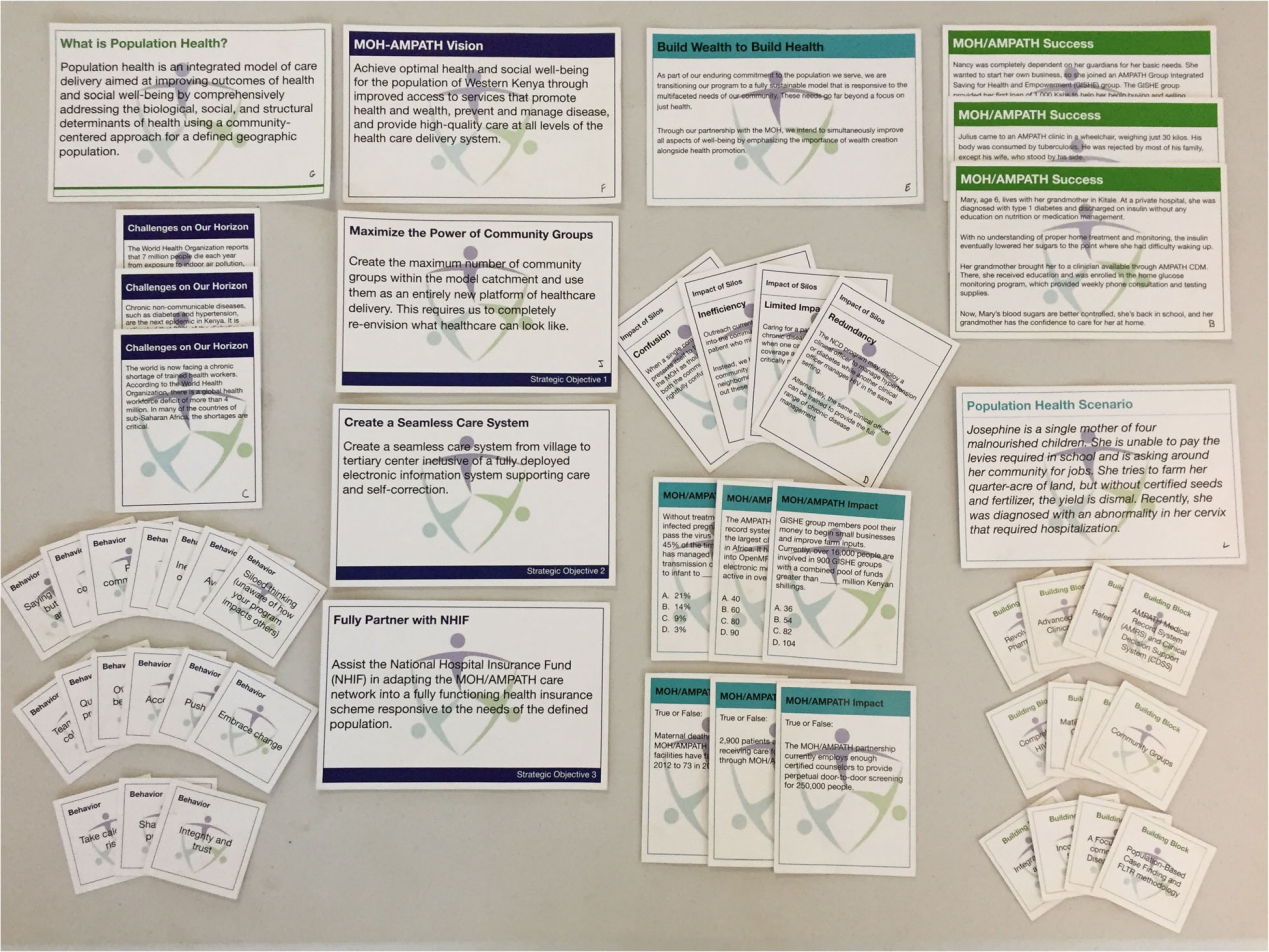
The Population Health Learning Map Experience

The facilitated discussion guide that accompanies the Learning Map® is broken into four main sections: 1) Building the Momentum; 2) Opportunities on the Horizon; 3) Daring to Dream; and 4) Making the Dream a Reality **(**Fig. 2**)**. Each of these sections is accompanied by a set of cards, including data elements, narrative stories, and questions to be read by participants that help facilitate the discussion, guiding groups of 8–10 participants through a 90-min experience, encompassing the vision of population health for western Kenya, and the strategy to achieve that vision.

**Fig. 3:**
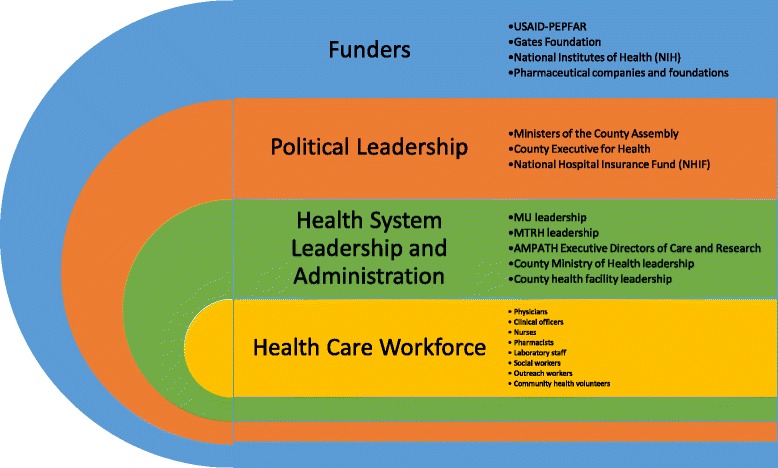
Population Health Learning Map delivered to stakeholders at all levels of the health care system

Once the Learning Map®, discussion guide, and cards were drafted, the Population Health Learning Map Experience was rolled out in Kenya over a one-week pilot period, including four sessions with senior leadership from the Ministry of Health, AMPATH, MTRH, MUCHS, as well as middle-management across the MOH-AMPATH care delivery system. These sessions served three purposes. First, was to present the vision for population health to key thought leaders and decision makers within the organization. Second, was to elicit feedback surrounding the delivery of the Learning Map®, and more importantly, the vision for population health and our strategy to implement it. Third, was to identify, and subsequently train, facilitators who can then lead staff across all levels of the MOH-AMPATH delivery system through the Learning Map® experience. During this pilot period, the messaging surrounding the Learning Map® was refined, however no major changes were made to the content. This reflected the fact that informal input was sought from many of these key thought leaders during the development phase, and in reality, AMPATH had been organically working towards this vision for many years. The key lesson in this Learning Map® Experience is that “the wisdom is in the room” and the map is just a metaphor, and a tool, for enabling people to share a common vision for change. Through this iterative process, AMPATH’s vision and strategy have crystallized, and we have created a core team of facilitators, or change agents, who are spreading the vision of population health throughout the pilot catchment area of 1 million people in western Kenya. Over the ensuing months, nearly 1000 MOH and AMPATH staff, of all different roles and cadres within the health system, have gone through the Population Health Learning Map® Experience. The Population Health Learning Map® has also been presented to politicians, sitting on the Health Committee of the local County Assembly, to galvanize their support for a robust, public sector, population health care delivery model in their constituencies. Lastly, the Population Health Learning Map® was presented to approximately 30 high-level executives from various pharmaceutical companies and foundations, the Gates Foundation, the National Institutes of Health, and USAID-PEPFAR during the AMPATH Partners Summit to share our vision with key international stakeholders and funders. In a short period of time, and by leveraging a unique set of collaborative partnerships, we have spread the vision for a comprehensive, integrated, community-centered, and financially sustainable model of population health to key stakeholders at all levels of the health system (Fig. 3**)**.

## Discussion

In order to implement a comprehensive, integrated, community-centered, and financially sustainable model of population health, AMPATH’s Population Health Care Delivery Model has three pillars:

1. Maximize the power of community groups
2. Create a seamless care system
3. Fully partner with NHIF

Implementation is initially occurring in two pilot sub-counties in western Kenya, and will eventually expand across all of AMPATH’s catchment area. Current efforts are focused on setting measurable goals and objectives, along with a specific and timely implementation work plan. In line with our commitment to partnerships and collaboration, a diverse group of AMPATH and MOH facility and health system leaders across multiple clinical departments are being brought together to coordinate efforts. Integration is occurring within facilities and across disease entities, where patients with HIV and chronic disease are being seen together, and providers are being cross-trained in multiple clinical disciplines. Similarly, integration efforts are occurring across levels of care with efforts focused on improving linkage to and retention in care, as well as strengthening referral networks across the health system. At the primary level, care is being decentralized to the community and layered onto existing microfinance groups, coupling economic development efforts with health care delivery, while ensuring enrollment in universal NHIF insurance cover. Simultaneously, a robust monitoring and evaluation, and quality improvement plan are being developed, allowing us to continually implement, measure, self-correct, and innovate on behalf of our patients and communities.

## Conclusion

The MOH-AMPATH population health care delivery model is a product of multiple collaborative partnerships. For 29 years, AMPATH – MU, MTRH, and a consortium of North American academic medical centers – have worked together with the Kenyan MOH to bring the depth and breadth of the tripartite academic mission to strengthen the public-sector health system in western Kenya. With the support of Dow AgroSciences and Root Inc., we leveraged expertise from the private sector in strategic planning and change management. This unique collaboration has allowed us to rapidly spread our vision for population health to key stakeholders at all levels of the health care system, including nearly every MOH-AMPATH health care provider in our population health catchment area of 1 million people across western Kenya. The Root Learning Map® module has become an essential tool to improve communication of the vision among all stakeholders, allow stakeholders to see the inter-relatedness of all partners involved in implementing the vision, create shared understanding of the vision, and enable each stakeholder to understand their role in relation to AMPATH’s strategic direction in population health. Through this process, our goal is that all stakeholders at all levels of the health system will have a renewed sense of ownership and commitment to implementing our vision of a comprehensive, integrated, community-centered, and financially sustainable population health care delivery model for western Kenya.

## Abbreviations

AMPATH: Academic Model Providing Access to Health Care
FP: Family Planning
MNCH: Maternal and Neonatal Child Health
MOH: Ministry of Health
MTRH: Moi Teaching and Referral Hospital
MU: Moi University
MUCHS: Moi University College of Health Sciences
NCDs: Non-Communicable Diseases
NHIF: National Hospital Insurance Fund
NIH: National Institutes of Health
PEPFAR: President’s Emergency Plan for AIDS Relief
pMTCT: Prevention of Mother-to-Child Transmission of HIV
TB: Tuberculosis
USAID: United States Agency for International Development
WHO: World Health Organization
